# Supplementary material for: Empirical methods for controlling false positives and estimating confidence in ChIP-Seq peaks
Source: BMC Bioinformatics. 2008 Dec 5;9:523. doi: 10.1186/1471-2105-9-523 (PMC2628906; doi:10.1186/1471-2105-9-523)
Supplement: Additional File 1 — A variety of html documents from the USeq web site detailing the available applications, their best usage, output file type descriptions, command line menus, etc. [file 1471-2105-9-523-S1.zip › USeqUserGuides/JavaDoc.zip!/JavaDoc/util/gen/Histogram.html]

Histogram


|  |  |  |
| --- | --- | --- |
| **Overview** \| **Package** \| **Class** \| **Use** \| **Tree** \| **Deprecated** \| **Index** \| **Help** | |  |
| **PREV CLASS**   **NEXT CLASS** | **FRAMES**    **NO FRAMES**     **All Classes** |  |
| SUMMARY: NESTED \| FIELD \| CONSTR \| METHOD | DETAIL: FIELD \| CONSTR \| METHOD |  |


---


## util.gen Class Histogram

```
java.lang.Object
  util.gen.Histogram
```

---

``` public class Histogram extends java.lang.Object ```

Generates histograms.

---

**Constructor Summary**

`Histogram(double[] scores,
int numberOfBins,
double tailTrimFraction)`
  
          Given an array of scores, creates a histogram of set number of bins, trims tails by a set fraction of the data.

`Histogram(double minimum,
double maximum,
int numberOfBins)`


| **Method Summary** | |
| --- | --- |
| `void` | `count(double value)`             Increments the proper bin. |
| `void` | `count(double value, double score)`             Increments the proper bin. |
| `void` | `countAll(double[] values)`             Counts all the values incrementing the proper bin. |
| `void` | `countAll(double[] values, double[] scores)`             Counts all the values incrementing the proper bin and scores. |
| `void` | `countAll(float[] values)`             Counts all the values incrementing the proper bin. |
| `int[][]` | `fetchCenteredFowardAndReverseCounts()`             Returns two columns as int[columns][counts] centered on max value with zeros as filler. |
| `int` | `findBinHits(double value)`             Given a value, finds the corresponding bin and returns the number of hits to that bin. |
| `int` | `findBinIndex(double value)`             Returns the index of the containing bin or -1 if value is too small or big. |
| `int[]` | `getBinCounts()` |
| `java.lang.String[]` | `getBinLabels()` |
| `double` | `getIncrement()` |
| `double` | `getMaximum()` |
| `double` | `getMinimum()` |
| `int` | `getNumberOfBins()` |
| `int` | `getNumLessThanMin()` |
| `int` | `getNumMoreThanMax()` |
| `int[]` | `getReversedBinCounts()` |
| `double` | `getTotalBinCounts()`             Including num less than and num more than. |
| `boolean` | `isMeanScoresPresent()` |
| `void` | `makeBins()` |
| `double` | `numberBinHitsToLeftAndIncludingValue(double value)`             Returns number of bin hits in the bins to the right of the value's bin including the numMoreThanMax. |
| `double` | `numberBinHitsToRightAndIncludingValue(double value)`             Returns number of bin hits in the bins to the right of the value's bin including the numMoreThanMax. |
| `double` | `numberBinHitsToRightOfValue(double value)`             Returns number of bin hits in the bins to the right of the value's bin including the numMoreThanMax. |
| `void` | `printBins()`             Prints bin info including mean if scores were provided while counting. |
| `void` | `printCenteredForwardAndReverseCounts()`             Prints two columns, forward counts and reversed counts centered on max value with zeros as filler. |
| `static void` | `printHistogram(int[] stars)`             Prints a simple histogram. |
| `static long` | `printHistogram(int[] realCounts, int[] stars, java.lang.String[] binLabels)`             Prints a simple histogram scaled using stars but real counts shown. |
| `static void` | `printHistogram(int[] stars, java.io.PrintWriter out)`             Prints a simple histogram to a PrintWriter. |
| `void` | `printScaledHistogram()`             Prints scaled histogram. |
| `double` | `pValue(double value)`             Returns a two tailed, p value given a threshold. |
| `double` | `pValueLeftSide(double value)`             Returns a one tailed, left side, p value given a threshold. |
| `double` | `pValueRightSide(double value)`             Returns a one tailed, right side, p value given a threshold. |
| `void` | `setIncrement(double increment)` |
| `void` | `setMaximum(double maximum)` |
| `void` | `setMinimum(double minimum)` |
| `void` | `setNumberOfBins(int numberOfBins)` |

**Methods inherited from class java.lang.Object**

`equals, getClass, hashCode, notify, notifyAll, toString, wait, wait, wait`

**Constructor Detail**

### Histogram

```
public Histogram(double minimum,
                 double maximum,
                 int numberOfBins)
```

---


### Histogram

```
public Histogram(double[] scores,
                 int numberOfBins,
                 double tailTrimFraction)
```

:   Given an array of scores, creates a histogram of set number of bins, trims tails by a set fraction of the data.
    Call printScaledHistogram() to print to screen.


**Method Detail**

### pValueRightSide

```
public double pValueRightSide(double value)
```

:   Returns a one tailed, right side, p value given a threshold.
    Assumes zero is base.
    Returns 1/total bin hits right side if value > last bin. Thus will not return zero.

---


### pValueLeftSide

```
public double pValueLeftSide(double value)
```

:   Returns a one tailed, left side, p value given a threshold.
    Assumes zero is base.
    Returns 1/total bin hits left side if value < last bin. Thus will not return zero.

---


### pValue

```
public double pValue(double value)
```

:   Returns a two tailed, p value given a threshold.
    Returns 1/total bin hits if value exceeds last bin. Thus will not return zero.

---


### numberBinHitsToRightOfValue

```
public double numberBinHitsToRightOfValue(double value)
```

:   Returns number of bin hits in the bins to the right of the value's bin
    including the numMoreThanMax.

---


### numberBinHitsToRightAndIncludingValue

```
public double numberBinHitsToRightAndIncludingValue(double value)
```

:   Returns number of bin hits in the bins to the right of the value's bin
    including the numMoreThanMax.

---


### numberBinHitsToLeftAndIncludingValue

```
public double numberBinHitsToLeftAndIncludingValue(double value)
```

:   Returns number of bin hits in the bins to the right of the value's bin
    including the numMoreThanMax.

---


### makeBins

```
public void makeBins()
```

---


### countAll

```
public void countAll(float[] values)
```

:   Counts all the values incrementing the proper bin.

---


### countAll

```
public void countAll(double[] values)
```

:   Counts all the values incrementing the proper bin.

---


### countAll

```
public void countAll(double[] values,
                     double[] scores)
```

:   Counts all the values incrementing the proper bin and scores.

---


### count

```
public void count(double value)
```

:   Increments the proper bin.

---


### count

```
public void count(double value,
                  double score)
```

:   Increments the proper bin.

---


### findBinIndex

```
public int findBinIndex(double value)
```

:   Returns the index of the containing bin or -1 if value is too small or big.

---


### findBinHits

```
public int findBinHits(double value)
```

:   Given a value, finds the corresponding bin and returns the number of hits to that bin.

---


### printScaledHistogram

```
public void printScaledHistogram()
```

:   Prints scaled histogram.

---


### printBins

```
public void printBins()
```

:   Prints bin info including mean if scores were provided while counting.

---


### printHistogram

```
public static long printHistogram(int[] realCounts,
                                  int[] stars,
                                  java.lang.String[] binLabels)
```

:   Prints a simple histogram scaled using stars but real counts shown.

---


### printHistogram

```
public static void printHistogram(int[] stars,
                                  java.io.PrintWriter out)
```

:   Prints a simple histogram to a PrintWriter.

---


### getReversedBinCounts

```
public int[] getReversedBinCounts()
```

---


### getTotalBinCounts

```
public double getTotalBinCounts()
```

:   Including num less than and num more than.

---


### getBinCounts

```
public int[] getBinCounts()
```

---


### getBinLabels

```
public java.lang.String[] getBinLabels()
```

---


### fetchCenteredFowardAndReverseCounts

```
public int[][] fetchCenteredFowardAndReverseCounts()
```

:   Returns two columns as int[columns][counts] centered on max value with zeros as filler.

---


### printCenteredForwardAndReverseCounts

```
public void printCenteredForwardAndReverseCounts()
```

:   Prints two columns, forward counts and reversed counts centered on max value with zeros as filler.

---


### printHistogram

```
public static void printHistogram(int[] stars)
```

:   Prints a simple histogram.

---


### getIncrement

```
public double getIncrement()
```

---


### setIncrement

```
public void setIncrement(double increment)
```

---


### getMaximum

```
public double getMaximum()
```

---


### setMaximum

```
public void setMaximum(double maximum)
```

---


### getMinimum

```
public double getMinimum()
```

---


### setMinimum

```
public void setMinimum(double minimum)
```

---


### getNumberOfBins

```
public int getNumberOfBins()
```

---


### setNumberOfBins

```
public void setNumberOfBins(int numberOfBins)
```

---


### getNumLessThanMin

```
public int getNumLessThanMin()
```

---


### getNumMoreThanMax

```
public int getNumMoreThanMax()
```

---


### isMeanScoresPresent

```
public boolean isMeanScoresPresent()
```


---


|  |  |  |
| --- | --- | --- |
| **Overview** \| **Package** \| **Class** \| **Use** \| **Tree** \| **Deprecated** \| **Index** \| **Help** | |  |
| **PREV CLASS**   **NEXT CLASS** | **FRAMES**    **NO FRAMES**     **All Classes** |  |
| SUMMARY: NESTED \| FIELD \| CONSTR \| METHOD | DETAIL: FIELD \| CONSTR \| METHOD |  |


---
